# Single-cell RNA sequencing reveals the role of immunoinflammatory cells in the progression of renal tubulointerstitial fibrosis

**DOI:** 10.1371/journal.pone.0337092

**Published:** 2025-11-21

**Authors:** Xiaoqin Ye, Youcai Xu, Shanshan Wu, Yu Peng, Liwen Gao, Xi Huang, Lingfei Lu, Jiandong Lu, Xinhui Liu

**Affiliations:** 1 Department of Nephrology, Shenzhen Traditional Chinese Medicine Hospital, Guangzhou University of Chinese Medicine, Shenzhen, Guangdong, China; 2 The Fourth Clinical Medical College, Guangzhou University of Chinese Medicine, Shenzhen, Guangdong, China; 3 Foshan Hospital of Traditional Chinese Medicine, Foshan, Guangdong, China; Brigham and Women's Hospital, UNITED STATES OF AMERICA

## Abstract

Renal tubulointerstitial fibrosis (TIF) is an independent risk factor for chronic kidney disease (CKD) progression and prognosis. It is known that immunoinflammatory cell infiltration plays a crucial role in TIF development and progression. However, what types of immunoinflammatory cells and by what means they promote TIF have not been fully clarified. In this study, mice models of unilateral ureteral obstruction (UUO) in which the left ureters were ligated for 3, 7, and 14 days, respectively, were used to simulate different levels of TIF severity. Single-cell RNA sequencing (scRNA-seq) was performed to characterize the immunoinflammatory cells in the kidneys of mice in each group. The results showed that the degree of renal pathological injury and expression level of fibrosis-related proteins increased over time in the UUO groups. Compared with the sham group, the proportion of T and NK cells, neutrophils, and mononuclear phagocyte cells was elevated in the kidney of UUO mice. Except for a decrease in the UUO7d group, the proportion of B cells did not differ notably between groups. The proportion of NaiveB_Ccl4 subset increased significantly in all UUO groups, and its up-regulated genes were mainly enriched in toll-like receptor signaling. The proportions of CD8Teff_Arhgap15, GDTCells_Trdc, HelperT_Tnf, and Treg_Foxp3 subsets were also significantly increased in all UUO groups, and their up-regulated genes were mainly enriched in NF-kappa B and TNF signaling. Neutrophils-4 subset was located at the terminal of neutrophil differentiation and mainly activated cytokine production and mitochondrial autophagy. Notably, the Macrophages_Arg1 subset had high scores in extracellular matrix remodeling, pro-angiogenesis, pro-inflammation, and immune regulation. Moreover, interactions between fibroblasts and immunoinflammatory cells increased with prolonged UUO time, with the strongest interactions with macrophages. When fibroblasts acted as ligand cells, the important interacting gene pairs with immunoinflammatory cells were CXCL6-CXCR1, APP-CD74, CX3CL1-CX3CR1, and THBS1-CD36, whereas when fibroblasts acted as receptor cells, the important interacting gene pairs with immunoinflammatory cells were TYROBP-CD44, TNF-TNFRSF1A, LGALS3-MERTK, PDGFA/B-PDGFRA, OSM-OSMR, and DKK2-LRP6. Overall, this study revealed the dynamic changes of immunoinflammatory cells and their interactions with fibroblasts in the kidneys during the UUO-induced TIF process.

## Introduction

Renal tubulointerstitial fibrosis (TIF) represents a prevalent pathological process in various chronic kidney diseases, posing significant challenges to global public health [1,2]. The pathogenesis of TIF is intricate, involving multiple cell types and molecular pathways. Recent studies have revealed that various cells within the renal tubule interstitium contribute to fibrosis progression [3–5]. The infiltration and activation of immunoinflammatory cells, including both tissue-resident and infiltrating cells from the innate and adaptive immune systems, are pivotal events in this process and key drivers of fibrosis progression [4,6]. These cells not only engage directly in the inflammatory responses but also amplify the local inflammatory microenvironment through the release of cytokines, chemokines, and other mediators, thereby exacerbating kidney tissue damage [7,8]. Additionally, fibroblasts transformation to myofibroblasts under the influence of various pro-inflammatory and pro-fibrotic factors further drives the development and progression of TIF [9]. Despite knowing the critical role of immunoinflammatory cell infiltration, the dynamic changes in immunoinflammatory cell types, their functional heterogeneity, and interactions with fibroblasts in the TIF process remain a major research focus.

Single-cell RNA sequencing (scRNA-seq) enables the rapid, simultaneous, and quantitative measurement of thousands of genes across thousands of single cells, offering an unprecedented opportunity to precisely define cell types and states at the molecular level [10]. Researchers have made substantial progress in characterizing acute kidney injury and chronic fibrosis using scRNA-seq [11,12]. In the present study, we established a unilateral ureteral obstruction (UUO) mouse model for 3, 7, and 14 days to simulate varying severities of TIF. scRNA-seq was employed to delineate the renal immunoinflammatory cell landscape across these time points. We characterized the dynamic changes, functional heterogeneity, and fibroblast interactions of immunoinflammatory cells throughout the TIF process. Our findings provide novel insights into immunoinflammatory mechanisms driving TIF and may inform the development of targeted therapeutic strategies.

## Materials and methods

### In vivo model

Twenty-four healthy 6-week-old male C57BL/6 mice were randomly divided into 4 groups after 1 week of adaptive feeding: sham group, 3-day UUO model group, 7-day UUO model group, and 14-day UUO model group, with 6 mice in each group. UUO was performed according to established procedures described in previous studies [13]. Briefly, each mouse was anesthetized with inhaled isoflurane (induction at 4%, maintenance at 1.5–2% in oxygen) and placed on a heated pad to maintain body temperature. Then the left ureter of the mice was exposed and ligated with 5.0 silk thread. To minimize suffering, the mice were monitored daily for signs of pain or distress (e.g., reduced mobility, weight loss > 20%). Any mouse exhibiting severe distress was scheduled for immediate euthanasia. Throughout the study, no such cases occurred requiring early euthanasia. For the sham group, the left ureters of mice were exposed but not ligated. For the UUO groups, mice were euthanized on the 3rd, 7th, and 14th days post-surgery via cervical dislocation under deep isoflurane anesthesia (5%), and then the unilateral kidneys were resected and weighed. Three kidney samples from each group were used for scRNA-seq analysis, and the remaining samples were stored for future use. All animal experiments were carried out in accordance with the U.K. Animals (Scientific Procedures) Act, 1986 and associated guidelines. Due to the limitations of our hospital’s animal experimentation facilities and long waiting time for appointments, we chose Shenzhen Glorybay Biotech Co., Ltd to conduct the animal experiments and obtained approval from the Ethics Committee (approved ID: RW-IACUC-22–0029).

### Western blot analysis

The renal cortex lysates were loaded in 7% SDS-PAGE gels for electrophoresis. Then the proteins were transferred to nitrocellulose membranes at a constant pressure of 100 V for 3 h. After blocking with 5% milk for 1 h, the membranes were incubated with primary antibodies against fibronectin (FN, ab2413), type I collagen (COL-I, ab6586, Abcam, Cambridge, United Kingdom), α-smooth muscle actin (α-SMA, A5228, Sigma-Aldrich, St Louis, MO, United States), vimentin (#5741), and α-Tubulin (#3873, Cell Signaling Technology, Beverly, MA, United States) at 4 °C in the refrigerator overnight. The next day the membranes were incubated with horseradish peroxidase-conjugated secondary antibodies at room temperature for 1 h. Then, the bands were visualized by the ChemiDoc™ MP Imaging System (Bio-Rad Laboratories, Hercules, CA, United States). Image Lab software version 5.1 (Bio-Rad Laboratories) was used to calculate gray values of the bands.

### Periodic acid-Schiff (PAS), Masson, and Sirius red staining

The kidney tissues were fixed in 4% paraformaldehyde, dehydrated with gradient alcohol, and embedded in paraffin. Kidney tissue wax blocks were cut into 4 μm sections and stained with PAS, Masson, and Sirius red. The representative staining images were captured by an Axio Imager M2 microscope and ZEN 2.6 software (Carl Zeiss, Jena, Germany). Tubular injury in PAS staining was scored according to the degree of tubular epithelial cell atrophy, detachment, and tubular dilatation. Scoring criteria were as follows: no renal tubular injury = 0; less than 10% = 1; 10%−25% = 2; 26%−50% = 3; 51%−75% = 4; greater than 75% = 5 [14]. The fibrosis area in Masson and Sirius red staining was calculated by using Image J software (NIH, Bethesda, Maryland, USA).

### Multiplex immunofluorescence staining

The tissue sections embedded in paraffin were fixed in 4% paraformaldehyde and then dewaxed to remove paraffin. Antigen retrieval was conducted using citric acid solution (PH 6.0) under high pressure. The sections were then placed in a light-proof wet box with 3% hydrogen peroxide for 25 mins at room temperature to inhibit endogenous peroxidase activity. Following this, 10% rabbit serum was applied for 30 mins at room temperature, after which sterile PBS mixed with the primary antibody was added and incubated overnight at 4°C. Once the sections were slightly dried, they were placed in the wet box again with the corresponding HRP-labeled secondary antibody and incubated for 50 mins at room temperature. After washing, tyramide signal amplification was added and incubated at room temperature in the dark for 10 mins. The bound primary and secondary antibodies were then removed using microwave treatment. This procedure was repeated three times from serum blocking to the incubation of the fourth antibody, followed by the addition of DAPI dye solution to stain the nuclei for 10 mins at room temperature, away from light. A self-fluorescence quenching agent was applied for 5 mins before sealing the slides, which were then imaged using a fluorescence microscope with an appropriate filter set.

### Preparation of single-cell samples

Fresh kidney tissues were preserved in sCelLive® Tissue Preservation Solution (Singleron Biotechnologies, Nanjing, China) within 30 min post-surgery. After washing three times with Hank’s Balanced Salt Solution, the tissues were minced. The tissue fragments were digested at 37 °C in the Singleron PythoN™ tissue dissociation system with 3 mL sCelLive® Tissue Dissociation Solution (Singleron Biotechnologies) for 15 min. The resulting cell suspension was filtered through a 40 μm sterile strainer. To remove red blood cells, GEXSCOPE® Red Cell Lysis Buffer (RCLB, Singleron Biotechnologies) was added to the suspension at a 1:2 volume ratio and incubated at room temperature for 5–8 min. The mixture was then centrifuged at 300 × g at 4 °C for 5 min, and the supernatant was discarded. The cells were resuspended in phosphate buffered saline (PBS).

### Construction of scRNA-seq library

Single-cell suspensions were diluted with PBS to a concentration of 2 × 10^5 cells/mL and loaded onto a microfluidic device. scRNA-seq libraries were constructed using the GEXSCOPE® Single Cell RNA Library Kits (Singleron Biotechnologies) according to the manufacturer’s protocol. The constructed libraries were diluted to 4 nM, pooled, and sequenced on an Illumina NovaSeq 6000 with 150 bp paired-end reads.

### Processing and analysis of scRNA-seq data

Raw reads were processed using CeleScope v1.5.2 (Singleron Biotechnologies) with default parameters to generate gene expression profiles. For quality control, dimensionality reduction, and clustering, Scanpy (version 1.8.2) under Python 3.7 was utilized, and cell clusters were visualized with uniform manifold approximation and projection (UMAP) in a two-dimensional space.

The raw sequencing reads were processed through a custom pipeline to produce gene expression matrices. Quality control was conducted using FastQC (version 0.11.4) and Fastp to remove low-quality reads, while Cutadapt was used for trimming poly-A tails and adapter sequences. Cell barcodes and UMIs were extracted from the reads, followed by alignment to the GRCm38 (mm10) reference genome using STAR software (version 2.5.3a). FeatureCounts (version 1.6.2) was then employed to obtain UMI and gene counts per cell, which were used to create expression profiles. Cells with UMI counts below 30,000, gene counts between 200 and 5,000, or mitochondrial content over 20% were filtered out. Dimensionality reduction and clustering were performed with the Seurat package in R (version 3.1.2), setting a resolution of 1.2 for sub-clustering specific cell types within a cluster. Visualization of cell subpopulations in two-dimensional space was achieved using the t-SNE or UMAP algorithm.

### Batch effect correction and differential expression analysis

Batch effects were corrected using Harmony (v1.0) applied to the top 20 principal components. Differential expression analysis was performed using the Wilcoxon rank-sum test in Scanpy. Genes were considered significantly differentially expressed if they were detected in >10% of cells in any group, exhibited an average log2(fold change) > 0.25, and had an adjusted p-value < 0.05 (Benjamini–Hochberg correction).

### Functional enrichment and cell annotation

Functional enrichment analysis of GO and KEGG pathways was conducted using clusterProfiler, with significantly enriched pathways defined as those with an adjusted p-value < 0.05. Cell types were annotated based on canonical markers from the SynEcoSysTM database. Subclustering of specific populations was performed to refine cell identities at higher resolution.

### Subtyping of major cell types

To obtain a high-resolution map of cell types, cells from the specific cluster were extracted and reclustered for more detailed analysis following the same procedures described above. The resulting cell clusters were annotated based on the expression of established canonical marker genes. Subclustering was then performed on major cell types (e.g., T cells, B cells) to identify finer populations. The names of these subclusters were assigned using a combination of two factors: (1) the broad, canonical cell type (e.g., NaiveB, CD8Teff) and (2) the expression of one or two highly specific and differentially expressed genes that characterize and distinguish that particular subcluster from others of the same lineage (e.g., Ccl4, Arhgap15). This naming convention aims to provide immediate information about both the cell’s identity and its unique functional state.

### Cell-cell interaction analysis

Cell-cell interactions between immunoinflammatory cells and fibroblasts were predicted based on known ligand–receptor pairs by CellphoneDB (v4.0.0). The permutation number for calculating the null distribution of average ligand-receptor pair expression in randomized cell identities was set to 1000. Individual ligand or receptor expression was thresholded by a cutoff based on the average log gene expression distribution for all genes across each cell type. Predicted interaction pairs with *P* value < 0.05 and of average log expression > 0.1 were considered significant and visualized by heatmap_plot in CellphoneDB [15].

### Statistical analysis

Data analysis and chart creation were conducted using GraphPad Prism 10 software (La Jolla, CA, United States). Data were presented as mean ± standard error of mean (SEM). Differences between groups were assessed using one-way ANOVA, followed by Tukey’s multiple comparison test for post hoc analysis. A *P* value of less than 0.05 was deemed statistically significant.

## Results

### Evaluation of TIF in UUO mice

**Fig 1:**
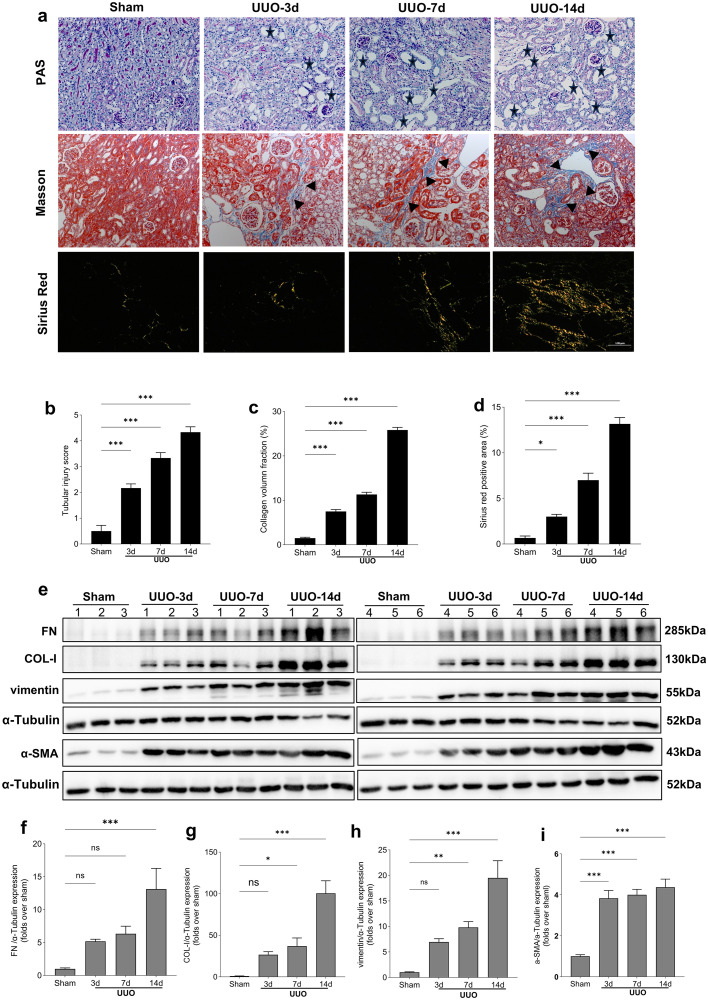
Evaluation of TIF in the kidneys of sham and UUO mice. (a) Representative PAS, Masson, and Sirius red staining images of kidneys from each group. All images are shown at identical magnification, × 200, scale bar = 100 μm. Pentagrams mark regions of tubular epithelial cell hypertrophy or degeneration. Triangles highlight areas of collagen deposition in the interstitium. Arrows quantify the level of collagen accumulation, and a higher density of arrows reflects more extensive fibrotic changes. (b-d) Quantitative analysis of tubular injury score and the degree of collagen deposition in different groups. (e) Representative Western blot images of FN, COL-I, vimentin, and α-SMA expression in the kidneys in each group. (f-i) Quantitative analysis of FN, COL-I, vimentin, and α-SMA normalized to α-Tubulin content. Data are expressed as mean ± SEM, n = 6 mice per group (ns = no significance, *P < 0.05, **P < 0.01, and ***P < 0.001 between the indicated two groups).

Periodic acid-Schiff (PAS) staining demonstrated that the renal tissue structure in the UUO groups was disordered, with atrophied and exfoliated renal tubule epithelial cells, dilated tubule lumens, and the severity of these pathological changes increased over time (Fig 1a and b). Masson staining revealed a time-dependent increase in the blue-stained areas, indicating collagen fiber deposition in the renal tissue of the UUO group compared to the sham group (Fig 1a and c). Furthermore, Sirius red staining also showed an increase in the red-stained collagen deposition in UUO mice (Fig 1a and d). Additionally, Western blot results confirmed that the expression levels of fibrosis-related proteins fibronectin (FN), type I collagen (COL-I), vimentin, and α-smooth muscle actin (α-SMA) increased over time in UUO mice (Fig 1e–i). These data collectively indicated that TIF progressively worsened with prolonged obstruction in UUO mice.

### Single-cell transcriptome landscapes of mice kidneys

**Fig 2:**
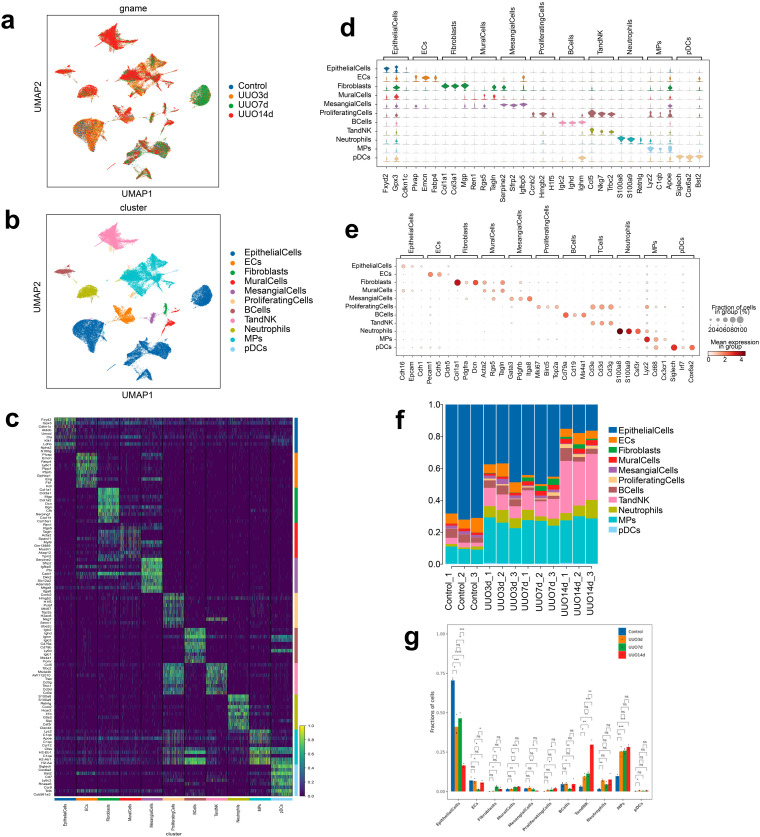
Single-cell transcriptome landscapes of mice kidneys. (a) Uniform manifold approximation and projection (UMAP) of renal cells colored by four groups. (b) UMAP of renal cells colored by 11 cell types. (c) Heatmap of the top 10 differentially expressed genes (DEGs) among all cell types according to log fold changes. (d) Violin plot of the top 3 DEGs among all cell types according to log fold changes. (e) Dot plot of the top 3 marker genes for all cell types. (f) Proportion of each cell type in each kidney sample. (g) Comparisons between groups of the proportion of each cell type in the total number of cells in the sample. Data are expressed as mean ± SEM, n = 3 (ns = no significance, *P < 0.05, **P < 0.01, ***P < 0.001, ****P < 0.0001 compared with indicated group).

Following sample preparation and quality control, we isolated a total of 102,273 cells for scRNA-seq analysis, with 19,919 cells from the control group, 35,366 cells from the UUO-3d group, 19,789 cells from the UUO-7d group, and 27,199 cells from the UUO-14d group (Fig 2a). Unsupervised cluster analysis allowed us to identify and characterize 11 cell types, including epithelial cells, endothelial cells (ECs), fibroblasts, mural cells, mesangial cells, proliferating cells, B cells, T and NK cells, neutrophils, mononuclear phagocyte cells (MPs), and plasmacytoid dendritic cells (pDCs) (Fig 2b). The heatmap of the top 10 differentially expressed genes (DEGs), the violin map of the top 3 DEGs, and the dot map of the top 3 cell marker genes for these 11 cell types were shown in Fig 2c–e, respectively. The proportions of the composition of these 11 cell types in each sample were illustrated in Fig 2f. Compared with the sham group, the epithelial cell proportion was significantly decreased in all UUO groups (*P *< 0.05), while the proportion of T and NK cells (*P *< 0.05) and MPs (*P *< 0.01) was obviously increased in all UUO groups. The increase in neutrophils was only significant in the UUO-3d group (*P *< 0.01) and fibroblasts were only significantly increased in the UUO-7d group (*P *< 0.05). B cells did not differ significantly between groups except for a decrease in the UUO-7d group (Fig 2g). These data provided an overview of the single-cell transcriptome of mice kidneys in each group.

### Characterization of B cells in TIF progression

**Fig 3:**
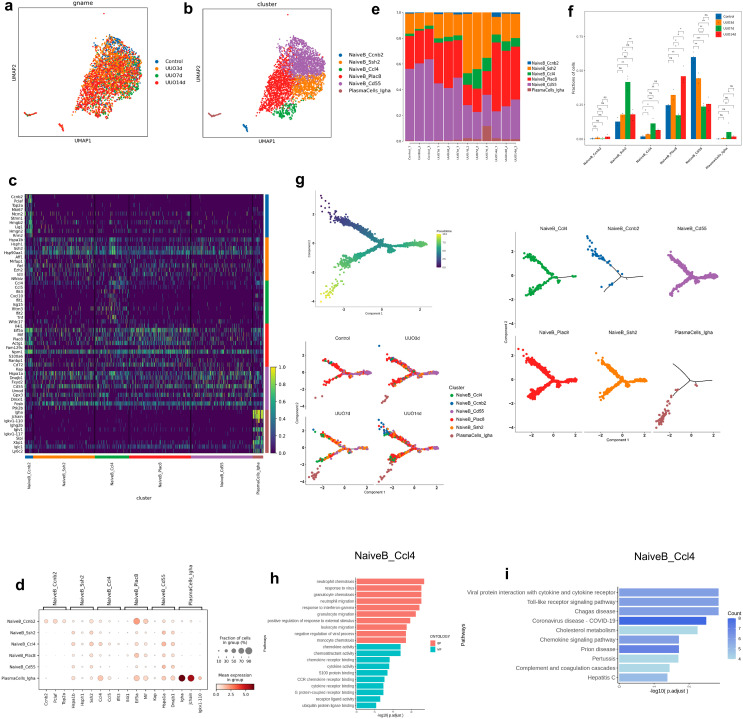
Characteristics of B cells transcription in the kidneys of sham and UUO mice. (a) UMAP of renal B cells colored by four groups. (b) UMAP of B cells colored by 6 cell types. (c) Heatmap of the top 10 DEGs among B cell types according to log fold changes. (d) Violin plot of the top 3 DEGs among B cell types according to log fold changes. (e) Proportion of each B cell type in each kidney sample. (f) Comparisons between groups of the proportion of each B cell type. Data are expressed as mean ± SEM, n = 3 (ns = no significance, *P < 0.05, **P < 0.01, ***P < 0.001 compared with the indicated group). (g) Pseudotime trajectory analysis of B cells, displayed by cell types and groups. (h) GO enrichment analysis of the upregulated genes of the NaiveB_Ccl4 subset. (i) KEGG enrichment analysis of the upregulated genes of the NaiveB_Ccl4 subset.

Subdividing B cells identified 6 distinct cell types: NaiveB_Ccnb2, NaiveB_Ssh2, NaiveB_Ccl4, NaiveB_Plac8, NaiveB_Cd55, and PlasmaCells_Igha (Fig 3a and b). The heatmap of the top 10 DEGs and violin map of the top 3 DEGs for these 6 cell types were shown in Fig 3c and d, illustrating their variations at the transcriptome level. The proportions of the composition of these 6 cell types in each sample were illustrated in Fig 3e. Particularly, the proportion of the NaiveB_Ccl4 subset increased significantly in all UUO groups (*P *< 0.05), while the proportion of NaiveB_Cd55 subset decreased with the prolongation of UUO (*P *< 0.01, Fig 3f). Pseudotime series analysis was performed to track the differentiation trajectory of each cell type. The NaiveB_Ccnb2 subset primarily resided at the beginning of the differentiation trajectory, while the PlasmaCells_Igha subset was predominantly at the terminal end (Fig 3g). The NaiveB_Ccnb2 subset was positioned as a precursor/reservoir state, while the accumulation of PlasmaCells_Igha represented the terminal outcome of B cell differentiation in the obstructed kidney. This active B cell lineage transformation highlights a significant adaptive immune response in renal fibrosis progression, wherein abundant antibodies (e.g., Igha) from plasma cells may promote local inflammation and tissue damage through mechanisms like immune complex deposition. GO and KEGG enrichment analyses of up-regulated genes of the NaiveB_Ccl4 subset revealed that pathways related to neutrophil chemotaxis, chemokine activity, and Toll-like receptor signaling were mainly enriched (Fig 3h and i). Overall, these findings revealed a concerted shift in B cell states during renal fibrosis, marked by the expansion of a pro-inflammatory NaiveB_Ccl4 subset—potentially recruiting innate immune cells via chemotaxis—and the terminal differentiation of antibody-secreting plasma cells. This implicated B cell-mediated humoral immunity as an active contributor to inflammatory tissue damage in obstructive nephropathy.

### Characterization of T and NK cells in TIF progression

**Fig 4:**
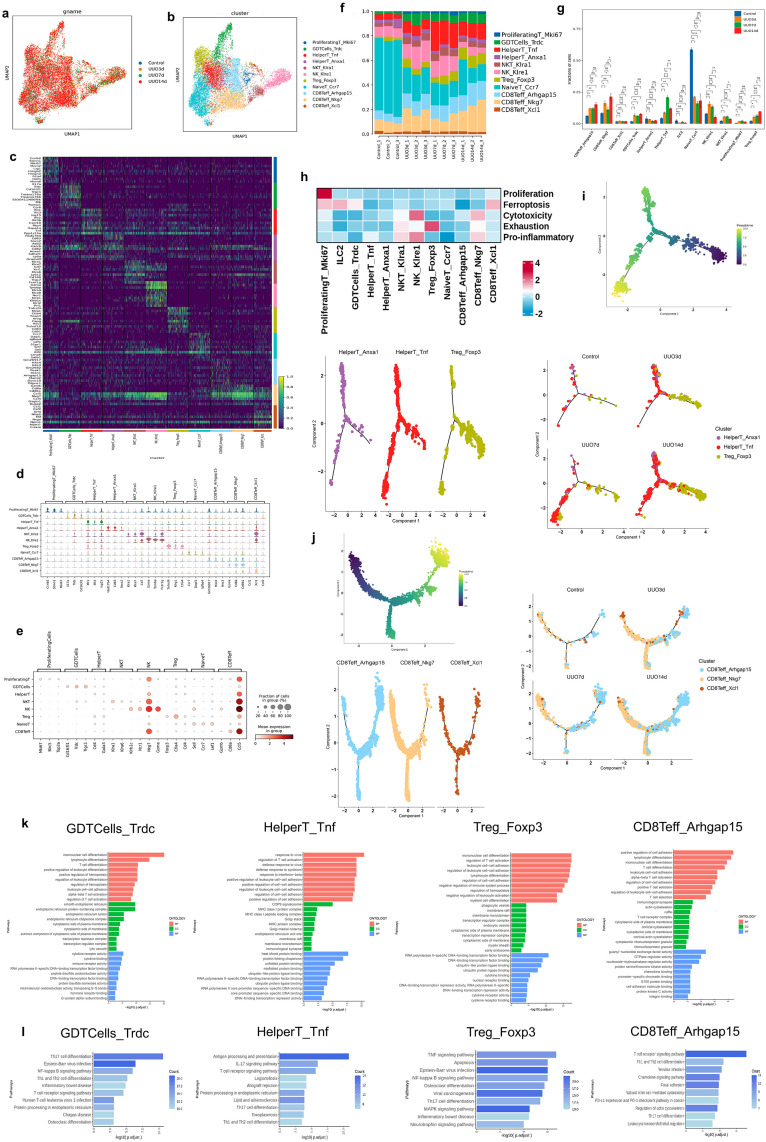
Characteristics of T and NK cells transcription in the kidneys of sham and UUO mice. (a) UMAP of T and NK cells colored by four groups. (b) UMAP of T and NK cells colored by 11 cell types. (c) Heatmap of the top 10 DEGs among T and NK cell types according to log fold changes. (d) Violin plot of the top 3 DEGs among T and NK cell types according to log fold changes. (e) Dot plot of the top 3 marker genes for all T and NK cell types. (f) Proportion of each T and NK cell type in each kidney sample. (g) Comparisons between groups of the proportion of each T and NK cell type. Data are expressed as mean ± SEM, n = 3 (ns = no significance, *P < 0.05, **P < 0.01, ***P < 0.001, ****P < 0.0001 compared with the indicated group). (h) Gene set scoring heatmap of all T and NK cell types. (i) Pseudotime trajectory analysis of CD4 + T cells, displayed by cell types and groups. (j) Pseudotime trajectory analysis of CD8 + T cells, displayed by cell types and groups. (k) GO enrichment analysis of the upregulated genes of the GDTCells_Trdc, HelperT_Tnf, Treg_Foxp3, and CD8Teff_Arhgap15 subsets. (l) KEGG enrichment analysis of the upregulated genes of the GDTCells_Trdc, HelperT_Tnf, Treg_Foxp3, and CD8Teff_Arhgap15 subsets.

By unsupervised clustering analysis, we identified 11 subsets in T and NK cells, including ProliferatingT_Mki67, GDTCells_Trdc, HelperT_Tnf, HelperT_Anxa1, NKT_Klra1, NK_Klre1, Treg_Foxp3, NaiveT_Ccr7, CD8Teff_Arhgap15, CD8Teff_Nkg7, and CD8Teff_Xcl1 (Fig 4a and b). The heatmap of the top 10 DEGs, the violin map of the top 3 DEGs and the dot map of the top 3 cell marker genes for these 11 cell types were depicted in Fig 4c–e. The proportions of the composition of these 11 cell types in each sample were illustrated in Fig 4f. Specifically, the proportion of the NaiveT_Ccr7 subset decreased (*P *< 0.05), while the abundances of the CD8Teff_Arhgap15 (*P *< 0.05), GDTCells_Trdc (*P *< 0.05), HelperT_Tnf (*P *< 0.01), and Treg_Foxp3 (*P *< 0.05) subsets increased in all UUO groups (Fig 4g). Additionally, a gene-set score heatmap was created for each cell type to elucidate their functional heterogeneity. The NK_Klre1 subset exhibited a high pro-inflammatory and cytotoxicity score, the Treg_Foxp3 subset displayed a high exhaustion score, and the ProliferatingT_Mki67 subset exhibited a high proliferation score (Fig 4h). Pseudotime analyses of the CD4+ and CD8 + compartments were shown in Fig 4i and j. Except for the Treg_Foxp3 subset, which was mainly located in the initiation segment of differentiation, there were no obvious differences in differentiation time of other subsets. We further performed enrichment analysis of the upregulated genes in 4 subsets that were increased in UUO mice. In GO analysis, cell adhesion, cell differentiation, cell activation, and cytokine/chemokine binding were the main enriched items (Fig 4k). Pathways of T cell receptor signaling, NF-kappa B signaling, and TNF signaling were common KEGG pathways (Fig 4l). In summary, our data characterized a transition towards an effector-driven T cell response in renal fibrosis. The upregulated NF-κB and TNF pathways in expanded subsets like CD8Teff_Arhgap15 and HelperT_Tnf functionally implicated them as key contributors to the sustained inflammation that directly fuels disease progression in UUO.

### Characterization of neutrophils in TIF progression

**Fig 5:**
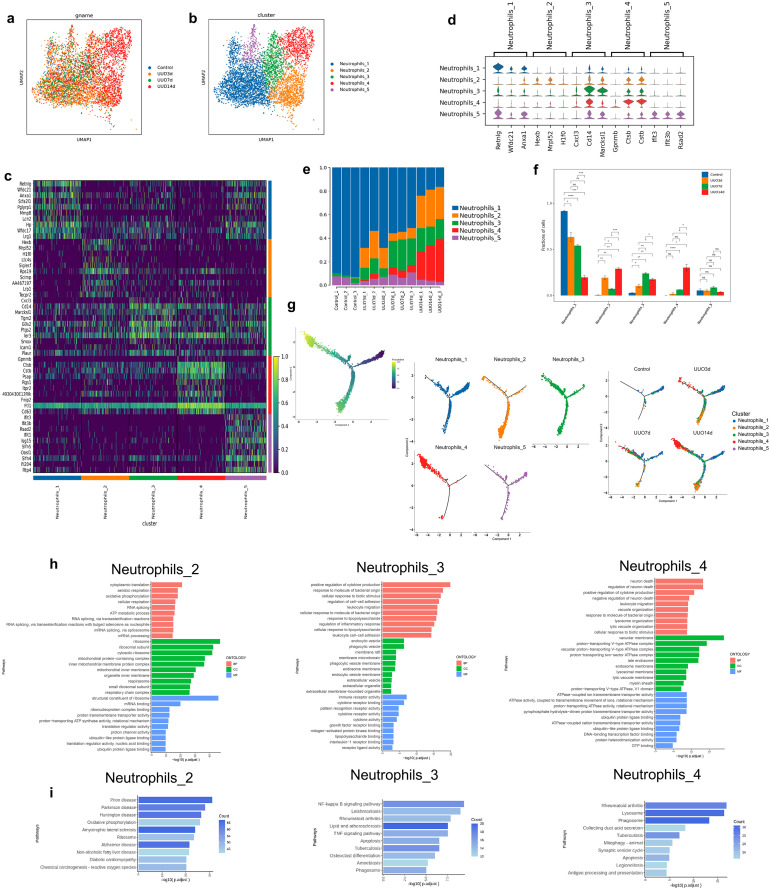
Characteristics of neutrophils transcription in the kidneys of sham and UUO mice. (a) UMAP of neutrophils colored by four groups. (b) UMAP of neutrophils colored by 5 cell types. (c) Heatmap of the top 10 DEGs among neutrophil types according to log fold changes. (d) Violin plot of the top 3 DEGs among neutrophil types according to log fold changes. (e) Proportion of each neutrophil type in each kidney sample. (f) Comparisons between groups of the proportion of each neutrophil type. Data are expressed as mean ± SEM, n = 3 (ns = no significance, *P < 0.05, **P < 0.01, ***P < 0.001, ****P < 0.0001 compared with the indicated group). (g) Pseudotime trajectory analysis of neutrophils, displayed by cell types and groups. (h) GO enrichment analysis of the upregulated genes of the Neutrophils_2, Neutrophils_3, and Neutrophils_4 subsets. (i) KEGG enrichment analysis of the upregulated genes of the Neutrophils_2, Neutrophils_3, and Neutrophils_4 subsets.

Subclustering the neutrophils resulted in 5 subsets: Neutrophils_1, Neutrophils_2, Neutrophils_3, Neutrophils_4, and Neutrophils_5 (Fig 5a and b). The heatmap of the top 10 DEGs and violin map of the top 3 DEGs for these 5 cell types were depicted in Fig 5c and d. The proportions of the composition of these 5 cell types in each sample were illustrated in Fig 5e. Monitoring the changes in proportions revealed a progressive decrease in the Neutrophils_1 subset, whereas the Neutrophils_2, Neutrophils_3, and Neutrophils_4 subsets were all increased in UUO mice (Fig 5f). Pseudotime analysis revealed that the Neutrophils_4 subset was located at the terminal differentiation stage (Fig 5g). Enrichment analyses of the upregulated genes in the Neutrophils_2/3/4 subsets found that cytokine production, NF-kappa B signaling, and apoptosis were the main enriched pathways (Fig 5h and i). These findings suggested that renal fibrosis was characterized by a dynamic shift in neutrophil states, with a loss of the homeostatic Neutrophils_1 subset and a concomitant expansion of pro-inflammatory (Neutrophils_2/3/4) subsets. The terminal differentiation state of Neutrophils_4 and its association with cytokine production and NF-κB signaling posit this subset as a key effector population that may perpetuate inflammatory injury and drive disease progression in the obstructed kidney.

### Characterization of macrophages in TIF progression

**Fig 6:**
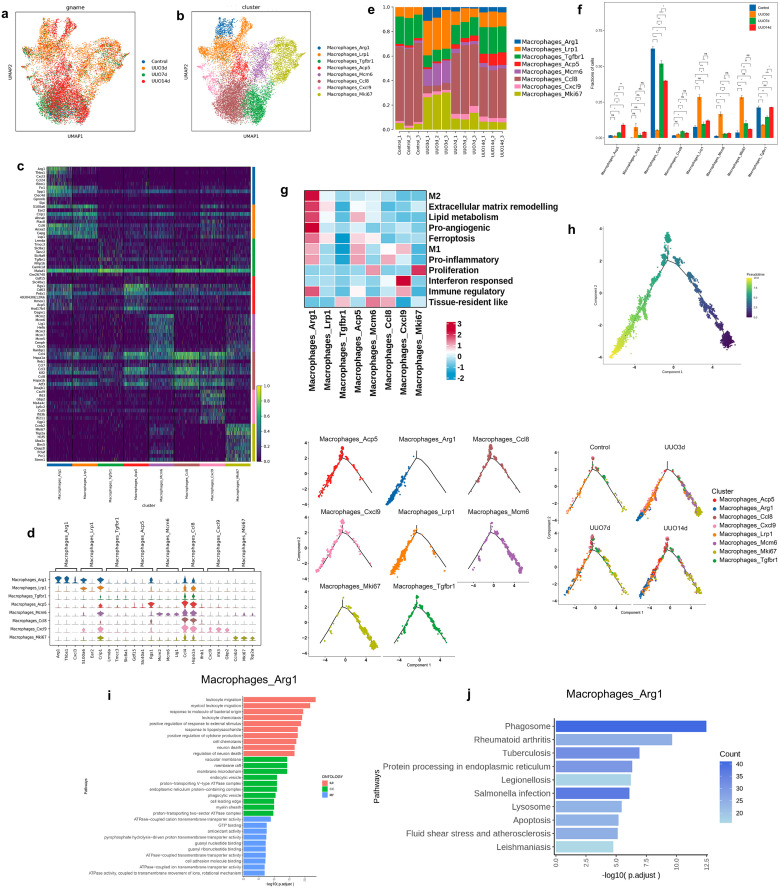
Characteristics of macrophages transcription in the kidneys of sham and UUO mice. (a) UMAP of macrophages colored by four groups. (b) UMAP of macrophages colored by 8 cell types. (c) Heatmap of the top 10 DEGs among all macrophage types according to log fold changes. (d) Violin plot of the top 3 DEGs among macrophage types according to log fold changes. (e) Proportion of each macrophage type in each kidney sample. (f) Comparisons between groups of the proportion of each macrophage type. Data are expressed as mean ± SEM, n = 3 (ns = no significance, *P < 0.05, **P < 0.01, ***P < 0.001, ****P < 0.0001 compared with the indicated group). (g) Gene set scoring heatmap of all macrophage types. (h) Pseudotime trajectory analysis of macrophages, displayed by cell types and groups. (i) GO enrichment analysis of the upregulated genes of the Macrophage_Arg1 subset. (j) KEGG enrichment analysis of the upregulated genes of the Macrophage_Arg1 subset.

Unsupervised cluster analysis of macrophages yielded 8 distinct cell types: Macrophages_Arg1, Macrophages_Lrp1, Macrophages_Tgfbr1, Macrophages_Acp5, Macrophages_Mcm6, Macrophages_Ccl8, Macrophages_Cxcl9, and Macrophages_Mki67 (Fig 6a and b). The heatmap of the top 10 DEGs and violin map of the top 3 DEGs for these 8 cell types were depicted in Fig 6c and d. The proportions of the composition of these 8 cell types in each sample were illustrated in Fig 6e. The proportion of the Macrophages_Acp5, Macrophages_Arg1, Macrophages_Cxcl9, Macrophages_Lrp1, Macrophages_Mcm6, and Macrophages_Mki67 subsets increased in UUO groups to varying degrees (Fig 6f). Notably, Ucell score heatmap revealed that the Macrophages_Arg1 subsetdemonstrated high scores for both M1 and M2 macrophages, as well as the highest scores in immune regulation, extracellular matrix (ECM) remodeling, pro-angiogenesis, and lipid metabolism (Fig 6g). Furthermore, pseudotime analysis illustrated the differentiation trajectory of macrophages, showing that the Macrophages_Arg1 subset was predominantly situated at the terminal of differentiation (Fig 6h). GO analysis of upregulated genes of the Macrophages_Arg1 subset showed that leukocyte migration, leukocyte chemotaxis, and cytokine production were mainly enriched (Fig 6i). Phagosome, protein processing in endoplasmic reticulum, lysosome, and apoptosis were mainly enriched KEGG pathways in the Macrophages_Arg1 subset (Fig 6j). These data revealed extensive macrophage heterogeneity in fibrotic kidneys. The expansion of multiple subsets, particularly the terminal Macrophages_Arg1 population, indicated a complex response that transcends conventional M1/M2 classification. With mixed M1/M2 markers and high ECM remodeling and immune regulation activity, this Arg1-high subset likely acted as a central orchestrator of inflammation, repair, and eventual fibrotic scarring in UUO.

### Characterization of fibroblasts in TIF progression

**Fig 7:**
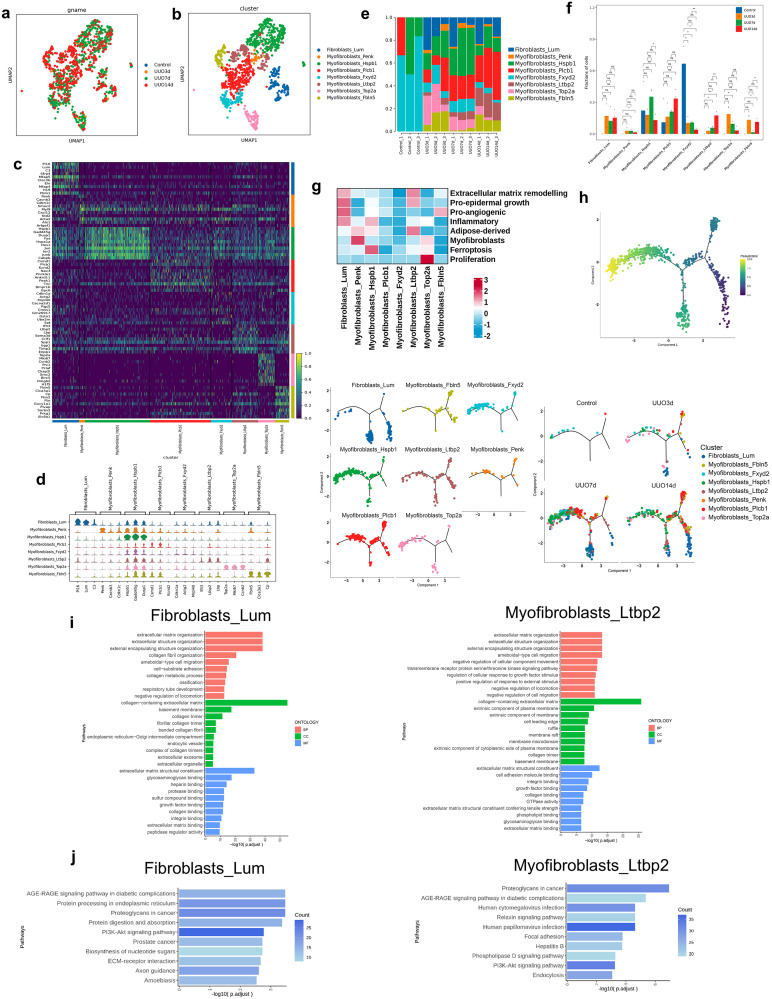
Characteristics of fibroblasts transcription in the kidneys of sham and UUO mice. (a) UMAP of fibroblasts colored by four groups. (b) UMAP of fibroblasts colored by 8 cell types. (c) Heatmap of the top 10 DEGs among all fibroblast types according to log fold changes. (d) Violin plot of the top 3 DEGs among fibroblast types according to log fold changes. (e) Proportion of each fibroblast type in each kidney sample. (f) Comparisons between groups of the proportion of each fibroblast type. Data are expressed as mean ± SEM, n = 3 (ns = no significance, *P < 0.05, **P < 0.01 compared with the indicated group). (g) Gene set scoring heatmap of all fibroblast types. (h) Pseudotime trajectory analysis of fibroblasts, displayed by cell types and groups. (i) GO enrichment analysis of the upregulated genes of the Fibroblasts_Lum and Myofibroblasts_Ltbp2 subsets. (j) KEGG enrichment analysis of the upregulated genes of the Fibroblasts_Lum and Myofibroblasts_Ltbp2 subsets.

Subclustering fibroblasts identified 8 cell types: Fibroblasts_Lum, Myofibroblasts_Penk, Myofibroblasts_Hspb1, Myofibroblasts_Plcb1, Myofibroblasts_Fxyd2, Myofibroblasts_Ltbp2, Myofibroblasts_Top2a, and Myofibroblasts_Fbln5 (Fig 7a and b). The heatmap of the top 10 DEGs and violin map of the top 3 DEGs for these 8 cell types were depicted in Fig 7c and d. The proportions of the composition of these 8 cell types in each sample were illustrated in Fig 7e. The proportion of Myofibroblasts_Plcb1 and Myofibroblasts_Ltbp2 subsets increased in a time-independent manner, but the proportion of Myofibroblasts_Fxyd2 subsets decreased in UUO mice (Fig 7f). The heatmap of gene set scores for each cell type was illustrated in Fig 7g. Notably, the Fibroblasts_Lum subset exhibited high scores for ECM remodeling, pro-epidermal growth, pro-angiogenic, and inflammatory. The Myofibroblasts_Ltbp2 subset also had high scores in ECM remodeling and pro-epidermal growth. In pseudotime analysis, the Fibroblasts_Lum subset was mainly localized in the initial and intermediate stages of differentiation, while the Myofibroblasts_Fxyd2 subset was mainly localized in the terminal stage (Fig 7h). GO and KEGG analyses revealed that ECM organization, extracellular structure organization, AGE-RAGE signaling, and PI3K-Akt signaling were most enriched in the Fibroblasts_Lum and Myofibroblasts_Ltbp2 subsets (Fig 7i and j). These results revealed a complex landscape of fibroblast activation in renal fibrosis. The expansion of ECM-remodeling subsets Fibroblasts_Lum and Myofibroblasts_Ltbp2, along with enriched PI3K-Akt and AGE-RAGE signaling, identified them as key drivers of pathological matrix deposition. Pseudotime analysis further suggested a differentiation trajectory from Fibroblasts_Lum toward terminal states, indicating its role as a progenitor-like population that initiates and sustains fibrosis.

### Interactions of immunoinflammatory cells with fibroblasts in TIF progression

**Fig 8:**
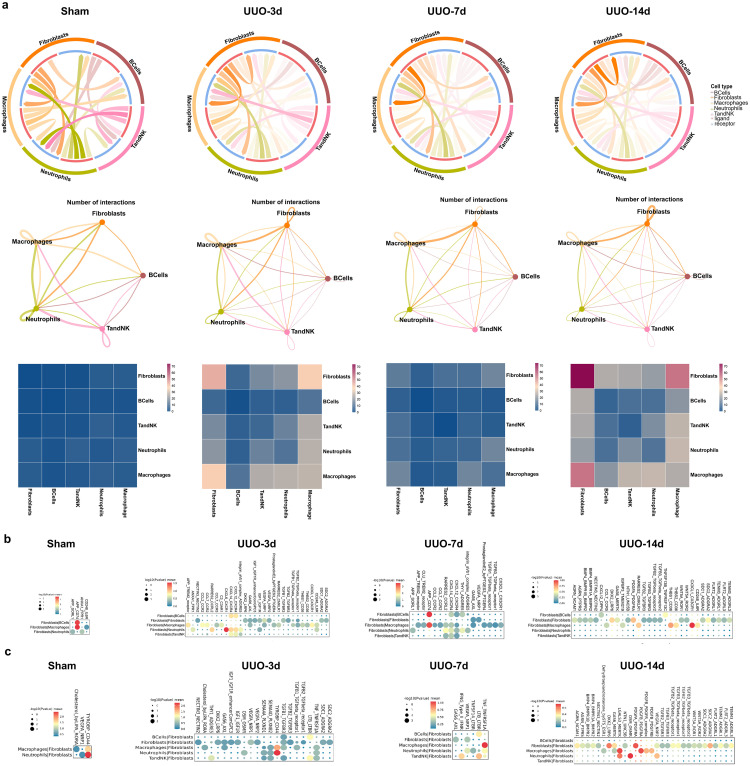
Interactions of immunoinflammatory cells with fibroblasts in the kidneys of sham and UUO mice. (a) Chord diagrams (top), network diagrams (middle), and heatmaps (bottom) of immunoinflammatory cells-fibroblasts interactions in each group. (b) The top 30 gene pairs in each group for immunoinflammatory cells-fibroblasts interactions. Fibroblasts acted as ligands, and immunoinflammatory cells acted as receptors. (c) The top 30 gene pairs in each group for immunoinflammatory cells-fibroblasts interactions. Immunoinflammatory cells acted as ligands, and fibroblasts acted as receptors.

**Fig 9:**
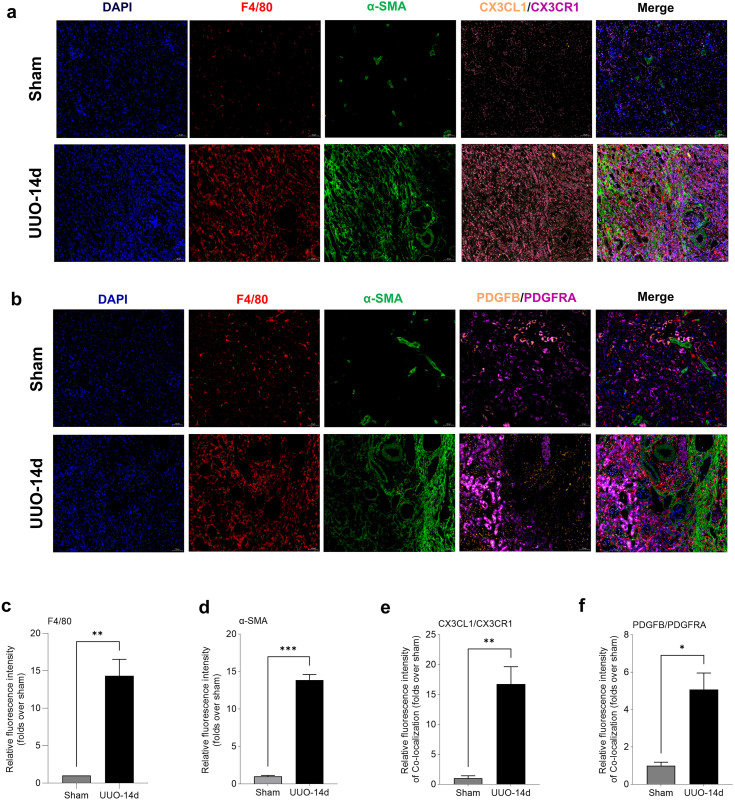
Multiple immunofluorescence staining and quantitative analysis of F4/80, α-SMA, CX3CL1/CX3CR1, and PDGFB/PDGFRA in the kidneys of sham and UUO-14d mice. (a) Representative immunofluorescence images of DAPI, F4/80, α-SMA, and CX3CL1/CX3CR1 in kidney tissue. (b) Representative immunofluorescence images of DAPI, F4/80, α-SMA, and PDGFB/PDGFRA in kidney tissue. All images are shown at identical magnification, × 200, scale bar = 50 μm. (c-f) Quantitative analysis of F4/80, α-SMA, CX3CL1/CX3CR1 co-localization, and PDGFB/PDGFRA co-localization in the sham group and UUO-14d group. Data are expressed as mean ± SEM, n = 3 mice per group (ns = no significance, *P < 0.05, **P < 0.01, and ***P < 0.001 between the indicated two groups).

We constructed an interaction network and heatmap to illustrate the intensity of interaction between immunoinflammatory cells and fibroblasts. The findings revealed that fibroblasts were crucial in regulating intercellular communication and exhibited strong interactions with immunoinflammatory cells, particularly macrophages. The network map and heatmap showed that the interaction between fibroblasts and macrophages was the strongest and reached a peak at 14 days post-UUO (Fig 8a). The top 30 gene interaction pairs between immunoinflammatory cells and fibroblasts were summarized in Fig 8b and c. In general, the UUO-3d and UUO-14d groups had the most interaction pairs, while the sham group had the least interaction pairs. We then focused on the UUO-14d group, which had the greatest degree of TIF. When fibroblasts acted as ligands, their interaction pairs with macrophages were the most numerous, with CX3CL1-CX3CR1, THBS1-CD36, and RARRES2-CMKLR1 being the most pronounced gene pairs. Conversely, when fibroblasts acted as receptor cells, the main gene pairs between macrophages and fibroblasts were LGALS3-MERTK and PDGFB-PDGFRA, and the main gene pairs between neutrophils and fibroblasts were OSM-OSMR and LGALS3-MERTK. The immunofluorescence co-staining of F4/80 (marker for macrophages), α-SMA (marker for fibroblasts), CX3CL1, and CX3CR1 and immunofluorescence co-staining of F4/80, α-SMA, PDGFB, and PDGFRA were shown in Fig 9a and b, respectively. Quantitative analysis indicated that the levels of F4/80 and α-SMA were significantly elevated in the UUO group compared to the sham group (Fig 9c and d). Moreover, the co-localization of CX3CR1 with CX3CL1, as well as PDGFB with PDGFRA, was particularly prominent in the UUO-14d group (Fig 9e and f). These data described the dynamic interactions between immunoinflammatory cells and fibroblasts in TIF and identified some important interacting gene pairs.

## Discussion

Our single-cell transcriptomic analysis revealed profound and dynamic alterations in the renal immunoinflammatory landscape during TIF progression**.** We identified distinct temporal trajectories and functional heterogeneity across four major immunoinflammatory cell types—B cells, T and NK cells, neutrophils, and macrophages—and uncovered critical interactions between these cells and fibroblasts. These findings provide novel insights into the cellular and molecular mechanisms driving TIF, highlighting potential immunomodulatory targets for therapeutic intervention.

Previous studies have shown that B cells can promote monocytes/macrophage recruitment and exacerbate tubular atrophy and fibrosis in the UUO model, ultimately impairing regeneration [16–18]. In our study, we observed a temporary decline in B cell numbers at 7 days, which we hypothesize may be due to an acute inflammatory response in the kidneys of UUO-7d mice, where macrophages and other inflammatory cells predominate. This environment may inhibit B cell function or disrupt their habitat. Additionally, local inflammatory mediators and cytokines might elevate B cell apoptosis, leading to their temporary reduction. Changes in chemokine expression during inflammation could drive B cells to migrate from the kidneys to secondary lymphoid organs like the spleen or lymph nodes, where they can respond to antigens and differentiate into plasma cells [16,19], thus not being included in our B-cell population analysis. This migration may be transient, explaining the increase in B cell numbers observed at 14 days. Furthermore, we found a significant rise in the proportion of the NaiveB_Ccl4 subset across all UUO groups, with its upregulated genes mainly enriched in toll-like receptor signaling pathways. This implies that B cells might play a role in TIF progression through innate immune mechanisms. Currently, the dynamic changes of B cells during renal fibrosis and their specific mechanisms remain unclear. We also described changes in the proportions of various T cell subsets (NaiveT_Ccr7, CD8Teff_Arhgap15, GDTCells_Trdc, HelperT_Tnf, Treg_Foxp3). Naive T cells, which are unexposed to antigens, may decrease in number as they differentiate into effector or memory cells involved in injury responses [20,21]. CD8 + effector T cells primarily function to identify and eliminate infected or abnormal cells [22]. The increased expression of Arhgap15, a GTPase-activating protein, may indicate enhanced cytotoxic activity, suggesting a stronger cell-mediated immune response to potential pathogens or damaged tissues [23]. However, in chronic inflammation, overactive CD8 + T cells can contribute to tissue damage and fibrosis, and both T cell receptor and NF-κB signaling pathways are integral to their activation and function, particularly during inflammatory responses [22]. The rise in GDTCells_Trdc (γδ T cells) might reflect a response to acute inflammation, but their prolonged presence could influence chronic inflammatory processes, thereby affecting fibrosis progression. γδ T cells respond quickly to various stimuli and can directly kill target cells, potentially increasing local inflammation through the release of adhesion molecules and cytokines [24]. Treg cells play a crucial role in tissue regeneration and repair, interacting with other immune repair cells like macrophages [25]. Research indicated that Treg cells not only expand in the early stages of kidney injury but also significantly increase later, corroborating our findings. Foxp3 is a pivotal transcription factor for regulatory T cells, which help suppress excessive immune responses and prevent autoimmune diseases [26]. While Tregs generally mitigate inflammation, they may also inadvertently support fibrosis in chronic conditions by inhibiting anti-fibrotic immune responses, exhibiting complex tissue-specific functions and plasticity [4]. The function of Tregs is linked to NF-κB and TNF signaling pathways, particularly in regulating inflammation, which our pathway enrichment analysis of this subset confirms. Overall, changes in T cell subsets create a complex immune microenvironment that balances pathogen defense and tissue repair with factors that can lead to persistent inflammation and fibrosis. Specifically, a disruption in the balance between pro-inflammatory cells (like CD8 + effector T cells and helper T cells) and anti-inflammatory/immunosuppressive cells (such as Tregs) may accelerate TIF. Furthermore, the enrichment of processes like cell adhesion, differentiation, and activation indicates that these T cell subsets not only undergo functional changes but also differ significantly in morphology and tissue localization, further influencing kidney structure and function.

Neutrophils are important effector cells in innate immunity [18]. In our research, we noted a gradual decline in the Neutrophils_1 subset, accompanied by an increase in the Neutrophils_2/3/4 subsets. We propose that the Neutrophils_1 subset is involved in the early stages of the acute inflammatory response. As fibrosis advances, other subsets become activated to execute various functions, including promoting inflammatory signaling, apoptosis, and cytokine production, while Neutrophils_1 migrate to the injury site to be either consumed or differentiate into other subsets. The rise in the three additional subsets indicates their potential significance in chronic inflammation or fibrosis, particularly the notable increase of the Neutrophils_4 subset at later stages, suggesting a more mature inflammatory state. This is further supported by extensive cytokine production, activation of NF-κB signaling, and apoptosis, which are crucial in the fibrosis process [27]. Regarding macrophages in renal fibrosis, substantial research has been conducted [1,28]. Our study revealed significant heterogeneity among macrophages in a UUO mouse model, identifying eight distinct subgroups, with the Macrophages_Arg1 subgset displaying unique traits. Arg1, a marker for M2-type macrophages, is typically linked to anti-inflammatory and tissue repair processes [29]. However, our findings indicated that the Macrophages_Arg1 subset exhibits characteristics of both M2 and M1 macrophages. Gene-set scores suggest this subset is involved in various immunomodulatory processes as well as ECM remodeling and angiogenesis, which are vital for sustaining chronic inflammation and fibrosis. Pseudotime analysis places Macrophages_Arg1 at the end of the differentiation pathway, indicating they have reached a highly differentiated terminal stage, potentially endowing them with specific functions. The up-regulated pathways identified in the GO/KEGG enrichment analysis underscore this subset’s significant role in the inflammatory response and tissue remodeling. Understanding these dynamic changes is crucial for elucidating the mechanisms of renal fibrosis and identifying new therapeutic targets.

In the present study, CX3CL1-CX3CR1 and PDGFB-PDGFRA were the most pronounced interaction gene pairs between fibroblasts and macrophages (Fig 8). CX3CL1 (Fractalkine) is unique among chemokines for its dual existence: as a soluble factor and as an adhesion molecule anchored to the cell surface. Its receptor, CX3CR1, is predominantly found on myeloid cells like monocytes and macrophages [30,31]. In fibrotic conditions, CX3CL1 facilitates the migration of inflammatory cells to injury sites and modulates local immune responses. It also plays a role in angiogenesis, potentially worsening tissue remodeling during repair. Research indicated that the CX3CL1/CX3CR1 axis contributes to the progression from chronic inflammation to fibrosis across various organs by activating resident fibroblasts and smooth muscle cells to deposit ECM [32–34]. Targeting this axis presents therapeutic opportunities for inflammatory or fibrotic diseases [35,36]. Although clinical-grade drugs directly targeting CX3CL1/CX3CR1 are limited, progress has been made with small molecule inhibitors and monoclonal antibodies. For instance, camel-derived single-domain “nano-antibodies” against CX3CR1 have been developed [37]. BI665088, a nano-antibody by Ablynx™ in partnership with Boehringer Ingelheim™, inhibited atherosclerosis development in human CX3CR1-expressing mice at 30 mg/kg. An anti-CX3CL1 monoclonal antibody showed promise for rheumatoid arthritis in early trials [38,39]. BMS-935177, an orally active small molecule inhibitor, selectively blocks CX3CL1/CX3CR1 interaction and reduces liver fibrosis in experimental NASH models. Clinical trials indicated a good safety profile for BMS-935177, though further research is needed to determine its efficacy in treating fibrosis [40]. Members of the platelet-derived growth factor (PDGF) family, including PDGFA and PDGFB, bind to the PDGF receptor α (PDGFRA) to initiate signaling that influences cell proliferation, migration, and survival. In fibrotic conditions, this pathway’s activation transforms fibroblasts into myofibroblasts [41]. PDGF also drives endothelial and smooth muscle cell transformation, promoting neovascularization and thus fibrosis progression. Several FDA-approved cancer drugs indirectly impact the PDGF pathway. For instance, imatinib, a tyrosine kinase inhibitor (TKI), has been applied to pulmonary fibrosis and other fibrotic diseases by blocking PDGF signaling and inhibiting fibroblast activity [42]. Crenolanib, an orally active small-molecule inhibitor targeting PDGF receptors’ α and β subtypes, originally developed for acute myeloid leukemia, has demonstrated preclinical efficacy in slowing fibrosis, notably in liver and kidney tissues [41,43]. Development is ongoing for monoclonal antibodies aimed at PDGF/PDGFR to target fibrosis more precisely without affecting normal tissue. These interventions could disrupt pro-fibrotic interactions between immune cells and fibroblasts, potentially slowing disease progression. The identified genes are crucial in fibrosis, offering potential therapeutic targets.

This study has several limitations. Firstly, while UUO mouse models are widely used for studying renal fibrosis, their pathological processes might not fully reflect those of human TIF, limiting the generalizability of our results. Secondly, our findings primarily stem from transcriptomic data. This study is descriptive and lack functional validation for key gene pairs and cell subsets. Future research should be validated through in vitro co-culture tests such as neutralizing antibodies or recombinant proteins, genetic perturbations of specific cell types (knockdown/knockout), and in appropriate in vivo disease models. Furthermore, while our scRNA-seq data robustly identified novel cell subsets such as *NaiveB_Ccl4* and *Macrophages_Arg1*, these populations were not independently validated by orthogonal methods (e.g., immunofluorescence or flow cytometry). The spatial localization and protein-level expression of subset-specific markers (e.g., CCL4, Arg1) remain to be confirmed. Critically, the proposed cellular interactions—such as the spatial proximity between Arg1 + macrophages and α-SMA+ myofibroblasts—were inferred from ligand-receptor pairing analysis but were not validated by spatial techniques (e.g., multiplex immunofluorescence). Finally, due to the different sensitivities of cell types to dissociation conditions, the proportion of cells observed in this study may not fully represent their true distribution in the original tissue. To make the single-cell sequencing results more reliable, this study set up independent biological replicates (n = 3), where all key findings were reproduced in the repeated samples, thereby effectively controlling the cell type ratio error caused by dissociation bias. Addressing these limitations can enhance our understanding of TIF mechanisms and offer new approaches for treating chronic kidney disease.

## Supporting information

S1 Fig Original Western blot strips. (PDF)
