# Supplementary material for: Single-cell RNA sequencing reveals the role of immunoinflammatory cells in the progression of renal tubulointerstitial fibrosis
Source: PLoS One. 2025 Nov 21;20(11):e0337092. doi: 10.1371/journal.pone.0337092 (PMC12637933; doi:10.1371/journal.pone.0337092)
Supplement: S1 Fig — (PDF) [file pone.0337092.s001.pdf]

Supplementary Raw Western Blot Data

Panel 1 represents western blot quantitative analysis shown in Fig 1e

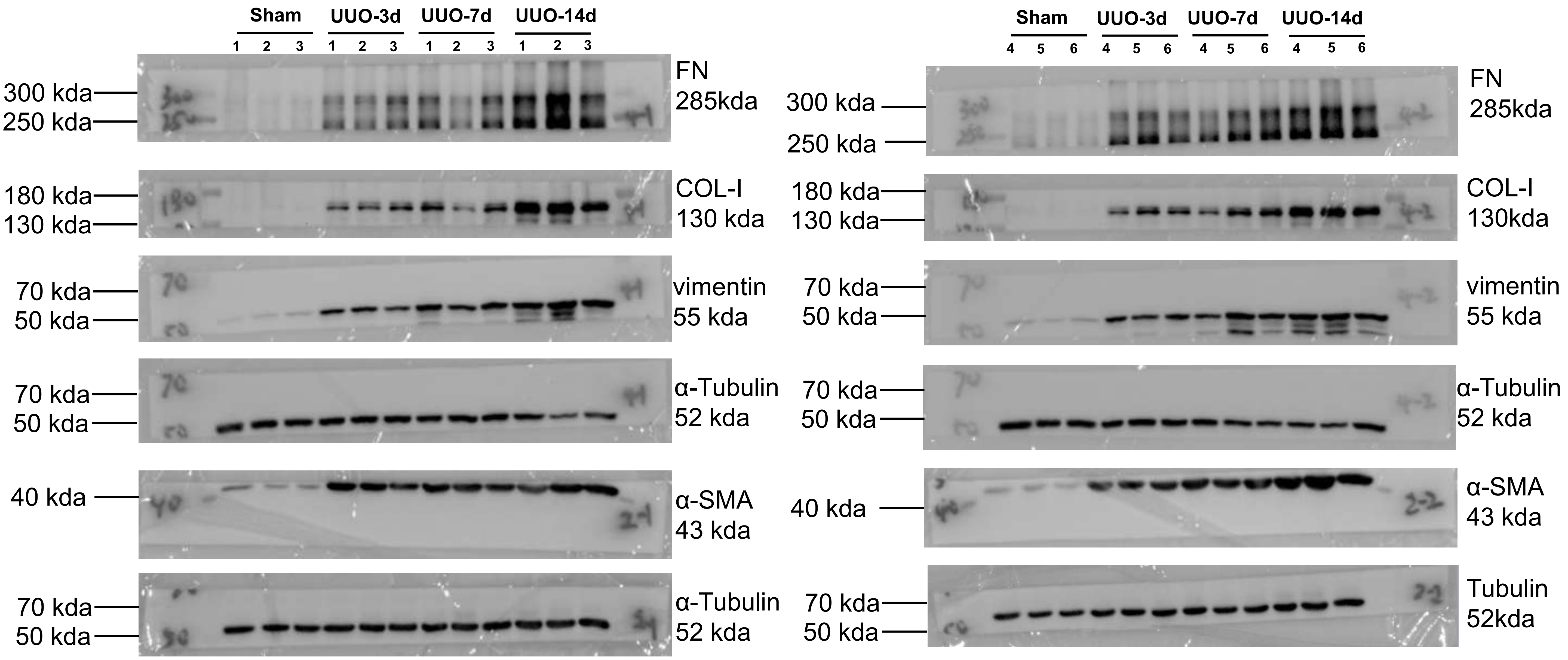

Marker: PageRuler™ Prestained Protein Ladder, 40 to 300 kDa, 26625, Thermo Scientific.
